# Sphincteroplasty for anal incontinence

**DOI:** 10.1093/gastro/gou003

**Published:** 2014-03-04

**Authors:** Lorenzo Carlo Pescatori, Mario Pescatori

**Affiliations:** Coloproctology Unit, Parioli Clinic, Rome, Italy.

**Keywords:** anal incontinence, sphincteroplasty, sphincter plication, pelvic floor repair

## Abstract

Sphincteroplasty (SP) is the operation most frequently performed in patients suffering from moderate-to-severe anal incontinence (AI) who do not respond to conservative treatment. Other costly surgeries, such as artificial bowel sphincter (ABS) and electro-stimulated graciloplasty, have been more or less abandoned due to their high morbidity rate. Minimally invasive procedures are widely used, such as sacral neuromodulation and injection of bulking agents, but both are costly and the latter may cure only mild incontinence. The early outcome of SP is usually good if the sphincters are not markedly denervated, but its effect diminishes over time. SP is more often performed for post-traumatic than for idiopathic AI. It may also be associated to the Altemeier procedure, aimed at reducing the recurrence rate of rectal prolapse, and may be useful when AI is due either to injury to the sphincter, or to a narrowed rectum following the procedure for prolapse and haemorrhoids (PPH) and stapled transanal rectal resection (STARR). The outcome of SP is likely to be improved with biological meshes and post-operative pelvic floor rehabilitation. SP is more effective in males than in multiparous women, whose sphincters are often denervated, and its post-operative morbidity is low. In conclusion, SP, being both low-cost and safe, remains a good option in the treatment of selected patients with AI.

## INTRODUCTION

Anal incontinence (AI) means the inability to control either gas or liquid faeces or solid stool, and affects a significant proportion of the elderly population—even if precise data are lacking, as most patients do not admit to suffering from this embarrassing disease. It may also follow ano-rectal surgery, e.g. internal sphincterotomy for anal fissure and low anterior rectal resection for cancer [1–3]. AI is caused by the alteration of one or more of the following factors: stool consistency, anal sphincter-, rectal reservoir- and pelvic floor innervation, which is mainly represented by the pudendal nerves. Their function is both sensorial and motor, as they allow both the perception of impending evacuation and the ability to retain the stool by eliciting the contraction of striated sphincters. The fact that nerve damage—e.g. from stretching during vaginal delivery—may cause AI explains why surgery alone may fail, and often a holistic approach is needed due to the altered quality of life [4].

## ALTERNATIVE PROCEDURES TO SPHINCTEROPLASTY

Dynamic graciloplasty may represent an alternative to sphincteroplasty (SP) but is costly, requires a covering stoma and carries high complication rate [5, 6]. It has therefore been almost abandoned. Gluteoplasty and artificial bowel sphincter (ABS) have been also used, but again their complication and failure rate is high [7–9].

Among the minimally invasive alternatives, sacral neuromodulation, first proposed by Matzel in 1995, is the only one that has the advantage that its outcome may be predicted by a provisional external stimulation [10]. Only if it works, the pacemaker will be implanted under local anaesthesia. The procedure, also used for some selected cases of constipation and proctalgia [11–13], carries a low post-operative morbidity and its outcome is successful in about 90% of cases in the long term [14].

Posterior tibial nerve electro-stimulation, first reported by Shafik *et al.* in 2003 [15], permits satisfactory results in patients with partial AI [16]; despite this, its benefit is questionable [17]. The stimulus, applied to the nerve in a peripheral location by means either needle or plaque electrodes, propagates upwards to the anal sphincters through the sacral nerves. The magnetic anal ring was popularized by Lehur *et al.* in 2010, who reported encouraging results using this expensive device [18]. Finally, radiofrequency energy—the so-called ‘Secca procedure'—may generate anatomically advantageous modifications in the anal canal, with contraction of the collagenous tissue followed by some degree of fibrosis. Felt-Bersma *et al.* reported good short-term results [19], but Kim *et al.* did not achieve similar positive outcomes and reported anal ulcerations [20].

In cases of localized internal sphincter defect, the injection of bulking agents—replacing the injection of autologous centrifuged fat—has been reported by Shafik and by our group [21, 22]. PTQ (silicone spherules), Durasphere (coated charcoal), Solesta, Coaptite and, more recently, Gatekeeper, have been reported as successful in cases of mild-to-moderate AI [23–30]. More recent reviews are less optimistic about the advantages of these products [31, 32].

## RECONSTRUCTION OF THE ANAL SPHINCTER

Sphincteroplasty, intended as sphincter reconstruction, has been widely reported by several authors in recent decades [33–37]. The short-term results are good (74% of improved continence at three months) but the long-term outcome is not satisfactory, decreasing to 48% at 80 months [38]. According to Wexner’s group, there is no difference in outcome if the SP is carried out by the overlapping technique or by means of a direct suture of the two divided ends of the sphincter [39]. It may be better to keep some fibrotic tissue at the two ends of the divided muscle, to ensure a stronger plasty. It is mandatory that the two divided ends of the external sphincters have an adequate blood supply and that the reconstructive suture is not under excessive tension, which of course would facilitate a dehiscence, with subsequent failure.

A modified surgical approach to women with obstetric anal sphincter tears, by separate suturing of the external and internal anal sphincter, has been reported by Lindqvist and Jernetz [40]. The outcome of sphincter reconstruction, as reported by the literature, is illustrated in Tables 1 and 2. More recently, a modified SP with the association of a biological porcine collagen mesh, aimed at reinforcing the reconstruction, has been reported as successful by Zutshi *et al.* [49], but their study reports short-term results in a small series.

**Table 1.** Results for overlapping sphincteroplasty: long term (<5 years)

Author	Year	No. of patients	Mean age	Positive outcome (%)
Morren *et al.* [52]	2001	55	39	56
Elton and Stoodley [53]	2002	20	NR	80
Tjandra *et al.* [54]	2003	23	45	74
Pfeifer [55]	2004	41	34	73
Martinez *et al.* [56]	2006	16	NR	87
Barisic *et al.* [38]	2006	65	NR	74

NR = not reported.

(Modified by Pelvic Floor Disorders. Santoro GA, Wieczorek AP, Bartram CI (eds.). Springer 2010)

**Table 2.** Results for overlapping sphincteroplasty: long term (>5 years)

Author	Year	No. of patients	Mean age	Positive outcome (%)
Karoui *et al.* [57]	2000	86	NR	49
Halverson and Hull [58]	2001	71	38.5	46
Buie *et al.* [59]	2001	191	37	62
Barisic *et al.* [38]	2006	65	NR	48
Maslekar *et al.* [60]	2007	64	NR	80
Soerensen *et al.* [61]	2008	22	31	50

NR = not reported.

(Modified by Pelvic Floor Disorders. Santoro GA, Wieczorek AP, Bartram CI (eds.). Springer 2010)

The main indication for sphincter reconstruction is represented by AI due to sphincter injury, e.g. following car or motorcycle accidents; sphincter lay-open, e.g. high wide fistulotomy; and obstetric traumas, e.g. incorrect ephysiotomy. According to our experience in over 1000 cases, traumatic AI is, together with congenital AI, the one with the higher AI score as compared with other aetiologies [50].

Troublesome prolonged vaginal deliveries of heavy babies in multiparous females may cause partial anterior disruption of both the internal and external sphincter. The muscle defect is easily detectable using transanal or trans-vaginal ultrasound with a rotating probe. In such cases, a reconstruction of both sphincters is likely to be more effective. According to Mahony *et al.* it is better not to constipate the patient after SP, as the straining required to evacuate hard stool may disrupt healing of the sphincter [51].

The post-operative complication rate after SP is not high, the most feared complication being suture dehiscence. Tables 3 and 4 illustrate the complication rate as reported by the literature.

**Table 3.** Results of post-anal repair: short term (<5 years)

Author	Year	No. of patients	Positive outcome (%)
Braun *et al.* [41]	1991	31	84
Briel and Schouten [42]	1995	37	46
Athanasiadis *et al.* [43]	1995	31	52
Matsuoka *et al.* [44]	2000	21	35

(Modified by Pelvic Floor Disorders. Santoro GA, Wieczorek AP, Bartram CI (eds.). Springer 2010)

**Table 4.** Results of post-anal repair: long term (>5 years)

Author	Year	No. of patients	Positive outcome (%)
Setti Carraro *et al.* [45]	1994	54	52
Riegel *et al.* [46]	1997	22	58
Abbas *et al.* [47]	2005	47	68
Mackey *et al.* [48]	2009	57	52

(Modified by Pelvic Floor Disorders. Santoro GA, Wieczorek AP, Bartram CI (eds.). Springer 2010)

Less frequently, a SP may be required to cure AI following internal sphincterotomy for anal fissure [1], or due to the anterior resection syndrome, in which three continence factors, i.e. anal sphincters, stool consistency and rectal reservoir, may be affected [2, 3, 62]. We occasionally needed to repair either a sphincter injury caused by PPH for hemorroids or a recto-vaginal setum injury following STARR for obstructed defecation [63, 64]. Sphincteroplasty—as either suturing of a sphincter defect or layered reconstruction—after high trans-sphincteric and recto-vaginal fistula’s excision has also been described [65–67].

## PLICATION OF PELVIC FLOOR MUSCLES

Sphincteroplasty intended as plication of pelvic floor muscle—the so-called ‘post-anal repair'—was invented by the late Sir Alan Parks [68]. Good functional results in a large series have been reported at St Mark’s Hospital by Browning *et al.* using this technique [69], but other authors could not replicate this successful outcome, possibly due to the denervation of the plicated muscles, which makes their contraction ineffective [44]. In such cases with denervated sphincters and altered rectal sensation, better results are achieved using sacral neuromodulation [70]. On the other hand, others have reported good results following post-anal repair when strict indications are followed [48]. A consensus paper by Altomare *et al.*, on the management of AI, confirms that the Parks’ procedure is still indicated in selected patients [71]. The aim of the operation is to elongate the anal canal and to narrow the ano-rectal angle, which is thought to be an important factor in AI.

Our personal experience with Parks’ post-anal repair has been positive in more than half of the patients. We prefer not to use non-absorbable sutures for plicating the muscles as suggested by Parks, because we had two cases of Prolene sutures migrating into the rectal lumen one month after surgery, which caused discomfort to the patients. The stitches had to be removed transanally in both cases [72, 73]. In our procedure, both external sphincter and pubo-rectalis muscles are plicated posteriorly and then an anterior levatorplasty is carried out. Results are good, but reports of large series and very long follow-up are lacking.

## SPHINCTEROPLASTY COMBINED WITH OTHER SURGERIES

SP intended as reinforcement of the pelvic floor following other surgeries—such as the Altemeier rectosigmoidectomy—has been widely described and is illustrated in Williams’ and Keighley’s textbook [74]. Wexner’s group reported reduced recurrence of prolapse when a levatorplasty is carried out [75], whereas more recently, others have suggested encircling the sigmoid colon above the colo-anal anastomosis using a porcine collagen biological mesh [76]. According to our experience, sphincteroplasty and mesh positioning minimizes the risk of descent into the pouch of Douglas, as both peritoneocele and enterocele may be associated with rectal procidentia, especially in hysterectomized women. Pelvic floor rehabilitation is likely to increase the success rate in patients operated upon with SP. It is usually carried out after the healing of the surgical wounds and consists of physiokinesitherapy or transanal electro-stimulation or biofeed-back [77].

To better assess the outcome of a procedure carried out for AI, together with the post-operative changes of AI grading and score, it is necessary to provide an evaluation of quality of life. Both the Rockwood and the GIQLI tests are suitable for such an evaluation [78, 79]. Anal incontinence rates at 5 years following SP are disappointing and adversely impact quality of life, yet do not appear to relate to sexual function [80].

## CONCLUSIONS

The management of AI is now less surgical than in the past, due to the fact that operations such as graciloplasty and ABS carry a high morbidity and achieve unsatisfactory long-term results. Less invasive procedures—such as Devesa’s peri-anal encirclement [81], the previously mentioned bulking agents and, above all, sacral neuromodulation—allow satisfactory results to be achieved with a low complication rate. However, the cost of the above-mentioned procedures ranges between €1000 and €15 000 per patient, whereas SP is not based on any device—unless it is reinforced with a biological mesh, which costs less than €500.

Therefore, it may be concluded that SP still has a positive role to play in the management of AI, as it carries a good outcome when pelvic floor rehabilitation is also performed, provided that strict indications are followed; the most important one being to avoid the operation in patients with dystrophic neuropathic sphincters.

**Conflict of interest:** none declared.
